# Public perception of the resumption of HPV vaccine recommendation in Japan: Twitter content analysis

**DOI:** 10.1093/heapro/daad153

**Published:** 2023-11-15

**Authors:** Marina Terada, Tsuyoshi Okuhara, Tomomi Nagasawa, Hiroko Okada, Eiko Goto, Takahiro Kiuchi

**Affiliations:** Department of Health Communication, Graduate School of Medicine, The University of Tokyo, Tokyo, Japan; Department of Health Communication, School of Public Health, Graduate School of Medicine, The University of Tokyo, Tokyo, Japan; Department of Health Communication, Graduate School of Medicine, The University of Tokyo, Tokyo, Japan; Department of Health Communication, School of Public Health, Graduate School of Medicine, The University of Tokyo, Tokyo, Japan; Department of Health Communication, School of Public Health, Graduate School of Medicine, The University of Tokyo, Tokyo, Japan; Department of Health Communication, School of Public Health, Graduate School of Medicine, The University of Tokyo, Tokyo, Japan

**Keywords:** HPV vaccine, social media, Twitter, health professionals, Japan

## Abstract

On 12 November 2021, after a stagnation of 8.5 years, Japan decided to resume proactive recommendations for HPV vaccines. However, it is not known how the public reacted to the resumption of proactive recommendations for HPV vaccines, which are key elements in health communication that promote HPV vaccination. This study aimed to capture discussions on HPV vaccination on Twitter and to identify user characteristics, sentiments, discussed themes and their potential reach before and after 12 November 2021, when the Ministry of Health, Labour, and Welfare’s decision to resume proactive recommendation for HPV vaccines was first reported by the media in Japan. This study conducted a content analysis of tweets related to HPV vaccines posted on 11 November and 12 November 2021. Tweets were coded in terms of user characteristics, sentiments (i.e. positive, negative and neutral) and themes. Reach was measured by the number of retweets, likes and followers. A total of 3623 tweets were identified. The results showed that approximately 50% of health professionals and researchers tweeted neutral content about HPV vaccines. The most frequently discussed theme was the safety and side effects of HPV vaccines, which was accompanied by mainly negative sentiments. Although health professionals and researchers are influential on Twitter, half of them tweeted neutrally about HPV vaccines. Influential professionals, such as health professionals and researchers, are expected to disseminate accurate information to correct misinformation and recommend HPV vaccination on Twitter to overcome the HPV vaccination crisis, which is characterized by the low vaccination rates.

**Contribution to Health Promotion Statement**

- Monitoring the spread of information about HPV vaccines, the users who disseminate, sentiments and themes discussed on Twitter, can be useful for understanding the public perception of HPV vaccines.
- Most Twitter users expressed neutral sentiments about HPV vaccines after news reports of Japan’s resumption of proactive recommendations. After the news reports, the most discussed theme was safety concerns regarding HPV vaccines, which were mainly tweeted negatively.
- Although health professionals and researchers gained more followers, approximately half of them tweeted neutrally about HPV vaccines. They are expected to disseminate accurate information and recommend HPV vaccination on Twitter.

## BACKGROUND

Human papillomavirus (HPV) is the most common sexually transmitted infection worldwide (Dunne *et al.*, 2007; Forman *et al.*, 2012; Kombe *et al.*, 2021). High-risk HPV types 16 and 18 account for approximately 71% of all cervical cancer cases (Crosbie *et al.*, 2013; de Martel *et al.*, 2017). Three types of HPV vaccines are approved: bivalent, quadrivalent and nine-valent; as of 2020 these vaccines were being introduced in 107 countries (Bruni *et al.*, 2021). The World Health Organization has called on a global strategy to eliminate cervical cancer and recommends that 90% of girls be fully vaccinated with HPV vaccines by the age of 15 (World Health Organization, 2020). Despite the highly effective prevention available (Arbyn *et al.*, 2018), vaccine coverage is still suboptimal globally and domestically (Gallagher *et al.*, 2018; Bruni *et al.*, 2021).

Japan reported one of the lowest HPV vaccine coverages among high-income countries (Bruni *et al.*, 2021) in fiscal year 2020, with an uptake of only 15.9% among eligible adolescent girls aged 12–16 years (The Ministry of Health, Labour and Welfare, 2022). In 2013, a national program for routine use of HPV vaccines was introduced with subsidies from local and national governments (Ikeda *et al.*, 2019). However, mass media repeatedly announced adverse effects of HPV vaccination and girls suffering from diverse adverse events (Ueda *et al.*, 2017; Okuhara *et al.*, 2019). The Ministry of Health, Labour and Welfare (MHLW) decided to suspend proactive recommendations for routine use of HPV vaccines which involved mailing leaflets and coupons to eligible individuals two months after the launch (The Ministry of Health, Labour and Welfare, 2013). Despite the continued availability of HPV vaccines through direct contact with local governments, this HPV vaccination crisis caused a sharp drop in coverage from a peak of approximately 70% among girls born between 1995 and 1999 to less than 1% among girls born after 2002 (Nakagawa *et al.*, 2020). Although previous studies suggest no evidence of an association between adverse events and HPV vaccination in Japan (Suzuki and Hosono, 2018), the suspension of proactive recommendation lasted for 8.5 years. On 12 November 2021, the MHLW decided to resume proactive recommendations for HPV vaccines, beginning in April 2022 (The Ministry of Health, Labour and Welfare, 2021). This news was reported by major media outlets, highlighting the need for strategic communication to increase vaccine coverage.

Twitter has been studied as a health research tool (Sinnenberg *et al.*, 2017), especially for monitoring vaccine-related discussions and measuring vaccine confidence and hesitancy (European Centre for Disease Prevention and Control, 2019; Puri *et al.*, 2020). Regarding information on HPV vaccines on Twitter, exposure to negative information about HPV vaccines influences users’ attitudes toward HPV vaccines, leading them to post negative opinions in English tweets (Dunn *et al.*, 2015). The proportion and trends of negative tweets about HPV vaccines compared to those of neutral and positive tweets have been studied mainly in English contexts (Dyda *et al.*, 2019; Luo *et al.*, 2019; Du *et al.*, 2020). Health professionals are encouraged to actively disseminate positive information about HPV vaccines through social media (Ortiz *et al.*, 2019), as provider communication is a strong facilitator of HPV vaccination (Oh *et al.*, 2021). More health professionals and organizations have begun to disseminate information in response to real-time HPV events (Massey *et al.*, 2018). In the United States’ HPV social media campaign, researchers identified and corrected misinformation while engaging in discussions on topics related to HPV vaccines. This campaign achieved high engagement, with over 370 000 total impressions and reaching over 33 000 individuals in ten weeks, highlighting the potential of social media campaigns for promoting HPV vaccines to a targeted audience (Sundstrom *et al.*, 2021). Thus, monitoring the spread of negative information about HPV vaccines, the users who disseminate and themes discussed on Twitter, can be a listening tool for understanding the issues of interest and concerns among the target population (European Centre for Disease Prevention and Control, 2019; Puri *et al.*, 2020).

Japan has the second largest number of users on Twitter, with 58 million users as of January 2022 (Statista, 2022). In 2020, 42.3% of people from all generations and a large proportion of HPV vaccine-eligible cohorts (69.0% and 69.7% of teens and 20s, respectively) used Twitter in Japan (Ministry of Internal Affairs and Communications, Japan, 2020). The study on Japanese tweets related to HPV vaccines analyzed 208 tweets from 2014 to 2017, found only 25 tweets containing accurate information, and the majority of tweets were labelled as ‘unknown’, with no specific discussion themes identified (Suzuki *et al.*, 2021). Furthermore, no study in Japan provides information about the characteristics of users who disseminate tweets regarding HPV vaccines, the sentiments of tweets (whether positive or negative), or the specific themes discussed. In particular, examining public reaction to the resumption of proactive recommendations for HPV vaccines after a long stagnation will improve understanding of current public attitudes toward HPV vaccines and help develop effective communication strategies to encourage HPV vaccination. The general aim of this study is to identify user characteristics, sentiments, discussed themes and their potential reach before and after 12 November 2021—the day it was reported that the MHLW had decided to resume proactive recommendations for HPV vaccination. Our research questions are as follows:

RQ1: Are there any specific characteristics of users (i.e. health professionals and researchers) who tweet about the HPV vaccine, and are there any differences in user characteristics before and after the news reports on the resumption of proactive recommendations for HPV vaccines?

RQ2: Is there any difference in the sentiment of the tweets before and after the news reports, and any difference in reach (i.e. the sum of followers) by sentiment after the news reports?

RQ3: Does the sentiment of tweets differ between users on Twitter? Is there any difference in reach (i.e. the sum of followers) by the type of users after the news reports?

RQ4: What are the most frequently discussed themes on Twitter regarding the HPV vaccine before and after the news reports and is there any difference in sentiment?

## METHODS

### Data extraction

The first author (MT) applied and was approved for academic research on the Twitter application programming interface (API) through a developer account (Twitter, 2022). Tweet data was obtained by using Python ver. 3.8.9 and Tweepy, a Python library for accessing the Twitter API.

To determine the study period, the daily tweet count and relative search volume were retrieved in Japanese Standard Time using the Twitter API and Google Trends, respectively. Twitter is utilized by 40% of the Japanese population (Ministry of Internal Affairs and Communications, Japan, 2020), while Google Trends boasts a broader usage, with 75.4% of the population (Statcounter, 2023). Therefore, we employed Google Trends to validate the upsurge in Google searches corresponding to days with a significant volume of tweets. We used the Japanese terms ‘*wakuchin*’ and ‘*yobouseshu*’ in the search, which correspond to the following English terms: ‘vaccine’, ‘vaccination’ and ‘immunization’. The Japanese refer to cervical cancer and HPV vaccines as ‘*shikyu keigan*’ and ‘*sikyu keigan wakuchin*’. We applied a language filter to retain only Japanese tweets and used the keywords (((HPV OR ‘*sikyu keigan*’) AND (‘*wakuchin*’ OR ‘*yobouseshu*’ OR ‘*yobou wakuchin*’)) OR Gardasil OR Cervarix OR Silgard) on Twitter and ‘*HPV wakuchin*’ on Google Trends based on previous studies (Okuhara *et al.*, 2018; Zhang *et al.*, 2020). Daily data were collected over seven months, which started approximately one month before 12 November 2021—the day media outlets reported that the MHLW had decided to resume recommending HPV vaccines, and ended one month after 1 April 2022—the day the proactive recommendation was initiated. This included two critical events related to HPV vaccination in Japan. As Supplementary Appendix 1 shows, a spike in interest was observed on 12 November 2021, which was reflected in tweets as well as on Google Trends. Therefore, we decided to analyze tweets from 11 November 2021 to 12 November 2021, just before and after the news report on resumption of proactive recommendations for HPV vaccines. All tweets that matched the search keywords on 11–12 November were extracted and manually coded for tweet data including user IDs, usernames, user descriptions, texts and the number of retweets, likes and followers.

### Data analysis

For RQ1, we manually classified users according to their usernames and self-descriptions, consistent with previous studies (Cole-Lewis *et al.*, 2015; Hand *et al.*, 2016; Keim-Malpass *et al.*, 2017). For RQ 2-3, guided by a previous study (Schonfeld *et al.*, 2021), sentiment analysis was conducted by classifying tweets into three categories: positive, negative and neutral. A positive label was assigned if the text indicated that users thought HPV vaccines were effective or safe; a negative label was given if the text indicated a belief that HPV vaccines were not safe, were ineffective, or that cervical cancer is not a serious disease and does not need an HPV vaccine to be prevented; and a neutral label was given otherwise or when the tweet was not clear and included no additional comment or information, such as hashtags. In these sentiment analyses, emojis and texts were analyzed. For RQ4, we classified the content of tweets into themes, with the coding unit being the chunk of words indicating the meaning of the tweets. For RQ4, our objective was to identify the most discussed themes quantitatively, leading us to initially employ deductive analysis for theme categorization (Kondracki *et al.*, 2002). From Japanese content analysis of online information about HPV vaccines (Okuhara *et al.*, 2018), we extracted five representative themes (side effects, suffering girls, biased mass media reports, preventable effects and cancer screening). This decision stemmed from the absence of previous studies defining discussed themes in Japan regarding HPV vaccines on Twitter, and Japan’s unique experience of an HPV vaccination crisis. Next, when discussions did not align with themes from previous studies, we conducted inductive coding, resulting in the application of both inductive and deductive approaches. Attachments such as shortened URLs and image hyperlink destinations were not analyzed because they were not tweets and therefore did not meet the purpose of this study. Subsequently, using the coding manual created by MT, the third author (TN) was trained to code 50 tweets. After training, approximately 20% (*n* = 725/3623) of the total sample of tweets was randomly selected, and TN coded them to assess inter-rater reliability for all categories. All data coding was performed using Microsoft Excel (version 16.66.1, Microsoft Inc.).

### Statistical analysis

We conducted descriptive analysis to assess the distribution of user categories, sentiments and themes. An additional descriptive analysis of retweets, likes and followers on 12 November 2021, was conducted to examine Twitter information interactions and reach (European Centre for Disease Prevention and Control., 2019). To identify influencers, a sub-analysis categorized health professionals, researchers and politicians into quartiles based on the number of followers. Users in the fourth quartile were defined as influencers, as they had the highest number of followers (over 85% in each user category). The impact of influencers was evaluated based on the number of retweets, likes and followers. The inter-rater reliability for user categories, sentiments and themes was calculated using the Gwet AC1 statistic, which is known for its consistency with low prevalence (Gwet, 2008; Nishiura, 2010; Wongpakaran *et al.*, 2013). Data analyses were performed using Python ver. 3.7.14 and R for macOS (v4.2.0, R Core Team 2022).

### Ethical issue*s*

This study used publicly available data and followed the Twitter developer policy. It was approved by the ethics committee of the Graduate School of Medicine of the University of Tokyo (2022197NI).

## RESULTS

### Sample description

A total of 3623 tweets were extracted, of which 349 were tweeted on 11 November 2021 and 3274 were tweeted on 12 November 2021. A total of 271 and 2657 unique users tweeted on 11 and 12 November, respectively. The number of tweets on 12 November was 9.38 times the number on 11 November.

### Characteristics of users who tweeted about HPV vaccines

Table 1 shows the definitions of user categories and the distribution of tweet volumes on 11 November 2021 and 12 November 2021 by user categories. Users were classified into the following categories: general public, mothers or fathers, health professionals (e.g. physicians and nurses), researchers, politicians, anti-science individuals, reputable news sources and bots. Users who could belong to more than one category were included in all the relevant categories (e.g. one user could be both a mother and a health professional). Inter-rater reliability of coding users ranged from 0.87 to 1.00. On both days, most of the tweets were from general public (approximately 80%), followed by mothers or fathers (approximately 5%) and health professionals (approximately 5%). The relative presence of unique anti-science users decreased from 5.8% on 11 November to 1.7% on 12 November.

**Table 1.** The definitions of user categories and the distribution of tweet volumes on 11 November and 12 November 2021 by user categories, *n* (%)

		11-Nov-21		12-Nov-21	
Category	Definition	Unique users	Tweet volume	Unique users	Tweet volume
General public	Twitter users without specific description can be categorized	221 (80.4)	285 (80.1)	2199 (80.2)	2614 (78.2)
Mothers or fathers	Mother, father or guardian	15 (5.5)	22 (6.2)	149 (5.4)	159 (4.8)
Health professionals	Physician, nurse, midwife, public health nurse, pharmacist, clinical laboratory technician, registered dietitian, physical therapist and dentist	12 (4.4)	19 (5.3)	154 (5.6)	211 (6.3)
Researchers	Professor, researcher, postdoctoral researcher and PhD	3 (1.1)	3 (0.8)	43 (1.6)	49 (1.5)
Politicians	Politician	3 (1.1)	3 (0.8)	41 (1.5)	51 (1.5)
Anti-science individuals	An account that asserts opposition to vaccines, COVID-19, the effectiveness of masks, and the science behind them, promoting conspiracy theories. (e.g. self-description such as ‘Vaccines are developed for population reduction’, etc.)	16 (5.8)	19 (5.3)	47 (1.7)	57 (1.7)
Reputable News sources	Mass media and News sources such as Japanese mass media (e.g. Yomiuri Shinbun, etc.)	1 (0.4)	1 (0.3)	39 (1.4)	69 (2.1)
Bots	Non-official news account, computerized bots providing news automatically	4 (1.5)	4 (1.1)	69 (2.5)	131 (3.9)
Total		275	356	2741	3341

^a^Each user can be categorized into more than one category therefore, the total number of users exceeds the total number of unique users.

^b^Percentages may not sum to 100 due to rounding.

### Sentiment of the tweets

Table 2 shows the sentiment distribution of tweets on HPV vaccines on 11 November and 12 November 2021 and indicators of interaction and reach on 12 November 2021. Inter-rater reliability of sentiment was 0.78. Example tweets within sentiment analysis included ‘HPV vaccine is effective at preventing cervical cancer and please make sure you receive it’ as positive label, ‘HPV vaccines killed many girls and they recommend it because of a profit for manufactures’ as negative label and ‘I have heard the MHLW resumed recommendations for HPV vaccines, but I am not sure if it is safe’ as neutral label. The proportion of positive tweets decreased from 44.7% on 11 November to 36.4% on 12 November. On 11 November, the volume of tweets with positive sentiment was the largest (44.7%); however, on 12 November, the volume of tweets with neutral sentiment became the largest (50.2%). The percentage of tweets with negative sentiment decreased from 27.8% on 11 November to 13.3% on 12 November.

**Table 2.** The sentiment distribution of tweets on the HPV vaccine on 11 November and 12 November 2021 and indicators of interaction and reach—retweets, likes and followers on 12 November 2021 (*n* = 3623)

	11-Nov-21	12-Nov-21	12-Nov-21					
	Sentiment n		Median of RT (IQR)	Sum of RT	Median of likes (IQR)	Sum of likes	Median of followers (IQR)	Sum of followers
Positive	156 (44.7)(%)	1193 (36.4)(%)	0 (0–0)	9627	1.0 (0–4.0)	40 350	331 (89–1130)	9 978 113
Neutral	96 (27.5)	1645 (50.2)	0 (0–0)	13 939	0 (0–2.0)	32 118	535 (154–2179)	36 169 806
Negative	97 (27.8)	436 (13.3)	0 (0–1.0)	2001	1.0 (0–4.0)	4573	424 (136.5––1264)	1 478 480
Total	349	3274						

^a^Percentages may not sum to 100 due to rounding.

^b^RT = retweets.

The total potential reach of these tweets (the sum of followers) was the highest at 36 169 806 users for neutral sentiment, followed by 9 978 113 users for positive sentiment and 1 478 480 users for negative sentiment.

### User categories and sentiment of tweets

Table 3 shows the distribution of user categories by sentiment on 11 November and 12 November 2021, and indicators of interaction and reach on 12 November 2021. On 11 November, 68.4% of tweets from health professionals were positive. However, this percentage decreased to 54.5% on 12 November, and 42.7% of tweets from health professionals were categorized as neutral. On 11 November, 66.7% of tweets from both researchers and politicians were positive, which decreased to 42.9% and 41.2%, respectively for the two categories on 12 November; the proportion of neutral tweets for these user categories on 12 November was 46.9% and 49.0%, respectively. On 12 November, 92.8% of tweets from reputable sources were neutral.

**Table 3.** The distribution of user categories by sentiment on 11 November 2021 and 12 November 2021, and indicators of interaction and reach—retweets, likes and followers on 12 November 2021, *n* (%)

	11-Nov-21			12-Nov-21			12-Nov-21					
User category	Positive	Neutral	Negative	Positive	Neutral	Negative	Median of RT (IQR)	Sum of RT	Median of likes (IQR)	Sum of likes	Median of followers (IQR)	Sum of followers
General public	128 (44.9)	80 (28.1)	77 (27.0)	972 (37.2)	1283 (49.0)	361 (13.8)	0 (0–0)	8810	0 (0–2.0)	25 091	374.0 (101.25–1169.75)	14 644 538
Mothers or Fathers	10 (45.5)	4 (18.2)	8 (36.4)	84 (52.8)	56 (35.2)	19 (11.9)	0 (0–0)	398	1.0 (0–5.0)	1956	318 (113.5–877)	375 051
Health professionals	13 (68.4)	6 (31.6)	0 (0)	115 (54.5)	90 (42.7)	6 (2.8)	1.0 (0–28.0)	9,960	8.0 (1.0–101.0)	38 688	2005 (307–26 261.5)	3 996 638
Researchers	2 (66.7)	1 (33.3)	0 (0)	21 (42.9)	23 (46.9)	5 (10.2)	0 (0–5.0)	1,041	3.0 (1.0–40.0)	4738	3348 (541–15 991)	816 058
Politicians	2 (66.7)	1 (33.3)	0 (0)	21 (41.2)	25 (49.0)	5 (9.8)	3.0 (1.0–12.0)	878	13.0 (4.0–30.5)	2373	2200 (726–7188)	1 092 016
Anti-science individuals	1 (5.3)	5 (26.3)	13 (68.4)	1 (1.8)	14 (24.6)	42 (73.7)	0 (0–1.0)	222	2.0 (0–5.0)	812	652 (186–1501)	368 706
Reputable News sources	1 (100)	0 (0)	0 (0)	1 (1.4)	64 (92.8)	4 (5.8)	8.0 (3.0–47.0)	4,773	10 (3.0–76.0)	7735	21 282 (75 844–349 548)	25 481 438
Bots	2 (50.0)	2 (50.0)	0 (0)	7 (5.3)	119 (90.8)	5 (3.8)	0 (0–0)	617	0 (0–0)	989	1021 (239–5264.5)	1 539 713

^a^Percentages may not sum to 100 due to rounding.

^b^RT = retweets.

The total potential reach among users (the sum of followers) was the highest for reputable news sources at 25 481 438, followed by 14 644 538 for general public and 3 996 638 for health professionals.

### Content of tweets

**Figure 1.**
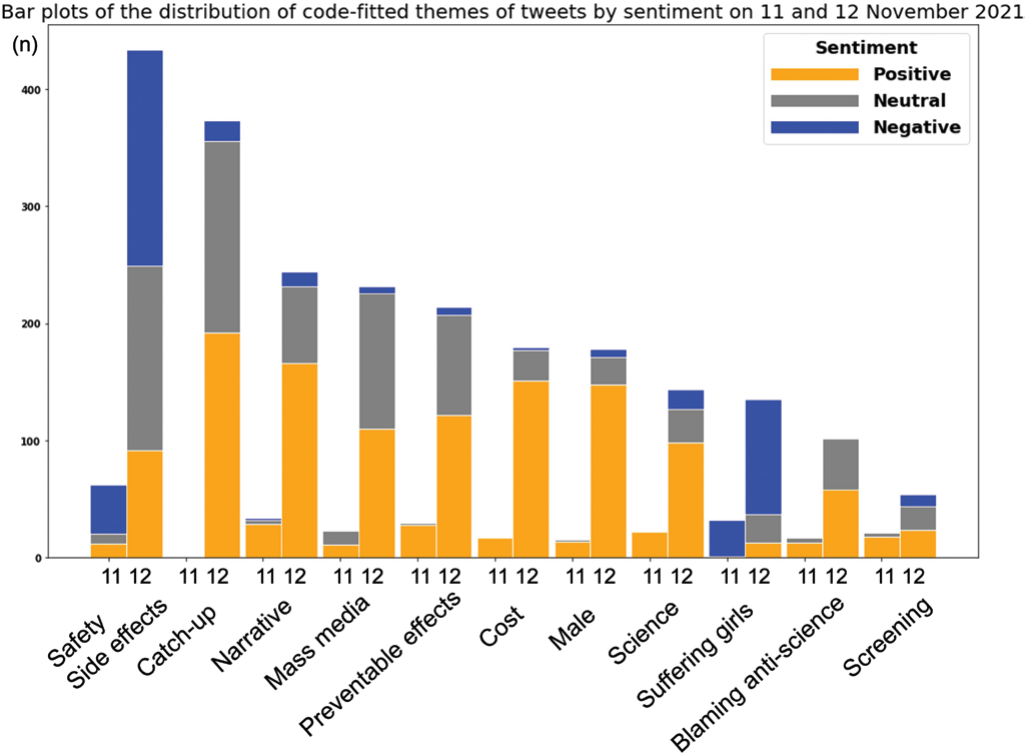
A graph depicting the distribution of code-fitted themes of tweets by sentiments on November 11 and 12, 2021. Orange indicates positive tweet volume, grey indicates neutral tweet volume and blue indicates negative tweet volume, respectively.

Table 4 shows the definitions, representative tweet examples and distribution of the code-fitted themes of tweets by sentiments on 11 November and 12 November 2021. Eleven themes were identified: ‘safety/side effects’, ‘cohorts missed opportunities of uptake’, ‘narrative’, ‘blaming biased mass media reports’, ‘preventable effects’, ‘cost’, ‘male’, ‘science’, ‘suffering girls’, ‘blaming anti-science’ and ‘screening for cervical cancer’. Inter-rater reliability of the coding themes ranged from 0.91 to 0.99. Figure 1 shows the distribution of code-fitted themes of tweets by sentiments on 11 November and 12 November 2021. On 11 November, the theme of ‘safety/side effects’ appeared most frequently (22.2%), followed by ‘narrative’ (12.2%) and ‘suffering girls’ (11.5%). On 12 November as well, ‘safety or side effects’ appeared most frequently (19.0%), followed by ‘cohorts missed opportunity of uptake’ (16.3%) and ‘narrative’ (10.7%). On 12 November, negative tweets included themes about ‘safety/side effects’ (42.6%), whereas positive and neutral sentiments varied across themes.

**Table 4.** The definitions, tweet examples and distribution of code-fitted themes of tweets by sentiments on 11 November and 12 November 2021, *n* (%)

Category	Definition	Tweet Examples	11-Nov-21				12-Nov-21			
			Positive	Neutral	Negative	Total	Positive	Neutral	Negative	Total
Safety/Side effects	Safety or side effects such as pain, swell and other symptoms	“The HPV vaccine is supposed to be an infertility drug. They are trying to make us get a dangerous vaccine, huh?”	12 (19.4)	8 (12.9)	42 (67.7)	62	92 (21.2)	157 (36.2)	185 (42.6)	434
Cohorts who missed opportunities of uptake	Cohorts born in FY1997-2006 who missed opportunity to receive the HPV vaccine due to the suspension	“Due to the negative media coverage during the timing when my sister and I could have received the HPV vaccine, we ended up not getting vaccinated after all.”	1 (50.0)	1 (50.0)	0 (0)	2	192 (51.5)	164 (44.0)	17 (4.6)	373
Narrative	Narrative of getting the HPV vaccine or getting cervical cancer without HPV vaccination	“I lost my sister to cervical cancer. I hope that eligible individuals consider getting vaccinated to protect themselves.”	29 (85.3)	3 (8.8)	2 (5.9)	34	166 (68.0)	66 (27.0)	12 (4.9)	244
Blaming biased mass media reports	Blaming biased mass media reports of adverse events of the HPV vaccine in 2013 or later	“It's quite surprising that TV media can broadcast such news, disregarding the extensive criticism of the "side effects" of the cervical cancer vaccine at that time in 2013. TV media should demonstrate whether their previous reporting was accurate or not.”	11 (47.8)	12 (52.2)	0 (0)	23	110 (47.4)	116 (50.0)	6 (2.6)	232
Preventable effects	Effectiveness of HPV vaccines to prevent cervical cancer and other sexual transmission diseases caused by HPV	“The HPV vaccine, which has been shown to prevent cervical cancer and other related diseases.”	28 (93.3)	2 (6.7)	0 (0)	30	122 (57.0)	85 (39.7)	7 (3.3)	214
Cost	Mention of cost and subsidies to receive HPV vaccines	“Has the cervical cancer vaccination campaign started? I received the Gardasil vaccine at my own expense. It was quite expensive.”	17 (89.5)	1 (5.3)	1 (5.3)	19	151 (83.9)	26 (14.4)	3 (1.7)	180
Male	Requesting the HPV vaccine to be eligible for male as well or mention of HPV vaccines effectiveness for male	“Indeed, the HPV virus is a frightening virus that can also be a cause of throat cancer. That's why it's important for both men and women to proactively get vaccinated.”	14 (93.3)	1 (6.7)	0 (0)	15	148 (83.1)	23 (12.9)	7 (3.9)	178
Science	Scientific research about HPV vaccines	“A study reports of no increase in side effects in comparison between vaccinated and unvaccinated individuals, both domestically and internationally. However, the media coverage on this matter has been scarce.”	22 (95.7)	1 (4.3)	0 (0)	23	98 (68.1)	29 (20.1)	17 (11.8)	144
Suffering girls	Mention of girls who suffered from side effects and who had been reported by media around 2013	“There are people who have received the cervical cancer vaccine and experienced symptoms such as pain in their limbs and body, fainting, walking difficulties, memory issues, etc., leading to their inability to attend school or being confined to a wheelchair for daily life.”	1 (3.1)	0 (0)	31 (96.9)	32	13 (9.6)	24 (17.8)	98 (72.6)	135
Blaming anti-science	Blaming anti-movement that increased vaccine hesitancy toward HPV vaccines	“It is disheartening to see how conspiracy theories and media influence have led to the removal of active promotion for the cervical cancer vaccine, resulting in harm to those affected by cervical cancer and their loved ones. I sincerely hope for a swift healing of their wounds and recovery for all those impacted by this situation.”	13 (76.5)	4 (23.5)	0 (0)	17	58 (56.9)	44 (43.1)	0 (0)	102
Cancer screening	Screening cytology or HPV test programs in Japan	“Absolutely, if the HPV vaccine can help prevent cancer, then it's definitely something worth getting vaccinated for. Even if regular screenings show no abnormalities, sometimes mild dysplasia can still be detected. Vaccination can provide an extra layer of protection and peace of mind in addition to routine check-ups.”	18 (81.8)	3 (13.6)	1 (4.5)	22	24 (44.4)	20 (37.0)	10 (18.5)	54

^a^Each user can be categorized into more than one category therefore, the total number of users exceeds the total number of unique users.

^b^Percentages may not sum to 100 due to rounding.

### Influencers

Supplementary Appendix 2 shows the distribution of sentiments of tweets among health professionals, researchers and politicians, as well as indicators of interaction and reach of the top influencers on 12 November 2021. The top 53 health professional influencers (4IQR) had 85.8% (*N* = 3 430 984) of all health professionals’ followers, with 49.1% tweeting positively, 45.3% tweeting neutrally and 5.7% tweeting negatively regarding HPV vaccines. The top 12 researcher influencers had 87.6% (*N* = 715 001) of all researchers’ followers, with 50.0% tweeting positively, 33.3% tweeting neutrally and 16.7% tweeting negatively regarding HPV vaccines. The top 13 politician influencers had 94.8% (*N* = 1 035 150) of all politicians’ followers, with 38.5% tweeting positively, 61.5% tweeting neutrally and 0% tweeting negatively regarding HPV vaccines.

## DISCUSSION

This content analysis presented an overview of the user characteristics, sentiments and themes of public discussions about HPV vaccines, and their potential reach on Twitter before and after the news reports of the resumption of proactive recommendations for HPV vaccines. The tweet volume of the HPV vaccine increased 9.4-fold after the news reports, indicating the magnitude of public interest.

Regarding user characteristics, general public who could not be classified into any specific category accounted for approximately 80% of users. Health professionals accounted for approximately 5% on both days which was lower than expected. Previous studies found that approximately 10% of English tweets related to HPV vaccines (Keim-Malpass *et al.*, 2017) stemmed from the medical community. Some health professionals may not explicitly mention their actual status on their profiles, leading to higher levels of anonymity. In future research, considering using ‘verified’ status to determine individual users’ actual status could be beneficial (Hernandez *et al.*, 2021). However, our study found that self-defined health professionals and researchers had higher median values for RTs and likes than other users. This finding suggests that users on Twitter may engage more actively, through retweets, likes or following, with users who self-identify as health professionals or researchers, indicating that health communication on social media by these professionals attracts more attention than that by lay individuals (Keim-Malpass *et al.*, 2017; Haman, 2020). This also suggests that users may perceive the status of others based on their usernames and self-descriptions. Furthermore, the proportion of anti-science users decreased following the news reports, possibly due to Twitter’s policy of regulating misinformation (Twitter, 2022) This policy may have suppressed anti-science activity on the platform. However, despite the decrease in anti-vaccine tweets, the user base on Twitter has doubled between 2015 and 2018, even regulation were in place (Gunaratne *et al.*, 2019). This suggests that individuals may resort to alternative, less public platforms to disseminate misinformation (Gunaratne *et al.*, 2019). In fact, the top anti-science user in this study with the most followers (over 200 000) did not mention negative misinformation in their tweets. Instead, the user included a URL and encouraged followers to transition to personal blogging. This indicates that negative misinformation about HPV vaccines may spread outside of Twitter. Future research can explore other platforms through URL destinations in tweets to evaluate the magnitude of the impact of anti-science activity.

The sentiment analysis showed that negative tweets were 27.8% before the news of the resumption of recommendation, which was similar to previous studies (e.g. 25–43%) (Dunn *et al.*, 2015; Keim-Malpass *et al.*, 2017; Du *et al.*, 2020). Previous negative media coverage prompted the MHLW to suspend the recommendation in 2013, resulting in Japan having the lowest vaccine confidence worldwide (de Figueiredo *et al.*, 2020). However, this study indicated that the news reports of resuming proactive recommendations for HPV vaccines might not elicit negative sentiment on Twitter during the study period.

Total reach, an indicator of public exposure based on sentiments, was the highest for neutral tweets, followed by positive and negative tweets. Thus, this study showed that exposure of the public to negative information was lower than anticipated. However, parental exposure to negatively biased information is associated with lower HPV vaccine initiation and delay (Margolis *et al.*, 2019). Exposure of the public to negative information on Twitter is an important indicator that should be carefully monitored. Given that the study period was limited, and negative tweets were expected to be posted more persistently than positive tweets (Bahk *et al.*, 2016), continuous monitoring is required.

Health professionals, researchers and politicians had more influence than other user categories because of their large numbers of retweets, likes and followers in this study. In addition, the sub-analysis showed that the influencers (those with the highest number of followers) among health professionals, researchers and politicians accounted for most of the followers. The median number of retweets and likes increased as the number of followers increased, suggesting that interaction and reach of Twitter discussions are strongly dependent on those influencers. Only half of the self-defined health professionals, researchers and politicians were positive towards HPV vaccines, whereas more than 40% (including influencers) were neutral. These results show a striking contrast with the results of a study reporting that 95.3% of advocacy users, including health professionals and research institutions, tweeted positively about HPV vaccines in English (Keim-Malpass *et al.*, 2017). In a previous study of Japanese physicians and nurses, only 35% of physicians and 11% of nurses recommended HPV vaccination for eligible adolescent girls (Nishioka *et al.*, 2022). The results of this study imply that health professionals took a passive stance on Twitter in addition to general practice, even after the announcement of the decision to resume proactive recommendations. Several studies have reported that recommendations by health professionals are persistent facilitators of HPV vaccination uptake by parents of adolescents and catch-up cohorts (Holman *et al.*, 2014; Loke *et al.*, 2017; Oh *et al.*, 2021). Providing peer-to-peer discussion and disseminating accurate information on social media by reputable and influential users, including health professionals and researchers, would be key to promoting HPV vaccination uptake.

Regarding the content of tweets, the most frequent theme was ‘safety and side effects’ (e.g. ‘HPV vaccine makes you infertile, and don’t forget the victims in wheelchairs who have difficulties walking!’). Negative sentiment dominated 42.6% of the tweets with the theme of ‘safety and side effects’. Side effects were the most common theme in Japanese online information on HPV vaccines in 2016 (Okuhara *et al.*, 2018). Concerns about safety and side effects are one of the barriers to HPV vaccination (Zheng *et al.*, 2021). Therefore, providing correct information regarding safety and side effects on Twitter is necessary to combat the propagation of negative misinformation.

This study had some limitations. First, Twitter data were extracted only from users whose Twitter profiles were set to public. However, Twitter accounts default to the public setting (Twitter, 2022), and the percentage of private accounts is considered small. Second, not all tweets are seen by all followers because not all users are active. Third, this study did not investigate the destination of URLs, images, and videos and may have lost some contextual information outside Twitter. However, 61.9% of tweets contained URLs, and 76.9% of those tweets cited news articles and the news titles and phrases were included in tweet texts for sentiment analysis and theme categorization. Fourth, the study period provided a limited view of public perception for a short period of two days. However, we found that the day after the study period ended, there was an approximate 50% drop in tweet counts; therefore, it is possible that the study captured public perception on the day of the reports to some extent. Fifth, this study estimated potential reach by using the sum of followers, which may result in duplication of users in sub-communities due to echo chambers. Thus, in future research, social network analysis can be conducted to measure the impact of each cluster. Sixth, this study demonstrated the perceptions of Twitter users; however, it did not encompass the perceptions of users of other social media platforms or the broader Japanese population. Seventh, this study applied only descriptive statistics; future research should examine whether exposure to Twitter information affects actual vaccine uptake. Despite these limitations, this study is the first to examine public reaction to the resumption of proactive recommendation for HPV vaccination in Japan following a long stagnation, and as mentioned earlier, it has important implications.

## CONCLUSION

This study explored public perception on Twitter on the resumption of proactive recommendation for HPV vaccines by the MHLW following an 8.5-year-long HPV vaccination crisis in Japan. This study found that approximately half of the health professionals, researchers and politicians (groups considered to be influential in promoting HPV vaccine uptake) tweeted neutrally about HPV vaccines. The most frequently discussed theme was concerned with the safety and side effects of HPV vaccines; it was mostly mentioned from a negative perspective. Influential professionals, including health professionals and researchers, are expected to provide peer-to-peer discussions and disseminate accurate information through tweets to correct misinformation and recommend HPV vaccination on Twitter and to contribute to overcome HPV vaccination crisis.

## Supplementary Material

daad153_suppl_Supplementary_Appendixs_1-2
