# Supplementary material for: Public perception of the resumption of HPV vaccine recommendation in Japan: Twitter content analysis
Source: Health Promot Int. 2023 Nov 15;38(6):daad153. doi: 10.1093/heapro/daad153 (PMC10647016; doi:10.1093/heapro/daad153)
Supplement: daad153_suppl_Supplementary_Appendixs_1-2 [file daad153_suppl_supplementary_appendixs_1-2.docx]

**Appendix 1. A graph depicting a tweet volume and Google Trends related the HPV vaccine.**

**Appendix 1. A graph depicting Google Trends data for the search word of the HPV vaccine in a blue line and absolute tweet volume data from Twitter API for the search word mentioned in the method paragraph in a red line. The highest volume of both data was the day MHLW reported to resume proactive recommendation for the HPV vaccine on November 12, 2021.**

| **Appendix 2.** The distribution of sentiments of tweets among health professionals, researchers and politicians, and indicators of interaction and reach of top influencers with highest followers on November 12, 2021 | | | | | | | | |
| --- | --- | --- | --- | --- | --- | --- | --- | --- |
|  |  |  |  |  |  |  |  |  |
|  |  |  |  |  |  |  |  |  |
|  | Quartile of followers | n | Positive | Neutral | Negative | Sum of followers | Median of RT | Median of likes |
| Health professionals | 1IQR | 53 | 26 (49.1) | 27 (50.9) | 0 | 7,279 (0.2) | 0 (0-1.0) | 0 (0-1.0) |
|  | 2IQR | 53 | 30 (56.6) | 22 (41.5) | 1 (1.9) | 40,850 (1.0) | 0 (0-1.0) | 2 (0-4.0) |
|  | 3IQR | 52 | 33 (63.5) | 17 (32.7) | 2 (3.8) | 517,525 (12.9) | 5.0 (1.0-34.75) | 26.5 (10.5-215.5) |
|  | 4IQR | 53 | 26 (49.1) | 24 (45.3) | 3 (5.7) | 3,430,984 (85.8) | 47.0 (18.0-95.0) | 236 (70.7-421.0) |
| Researcher | 1IQR | 13 | 7 (53.8) | 5 (38.5) | 1 (7.7) | 3,293 (0.4) | 0 (0-0) | 1 (0-2.0) |
|  | 2IQR | 12 | 2 (16.7) | 10 (83.3) | 0 | 14,870 (1.8) | 0 (0-0) | 0.5 (0-4.0) |
|  | 3IQR | 12 | 6 (50.0) | 4 (33.3) | 2 (16.7) | 82,894 (10.2) | 1.5 (0-3.25) | 5.5 (3.0-13.25) |
|  | 4IQR | 12 | 6 (50.0) | 4 (33.3) | 2 (16.7) | 715,001 (87.6) | 47.5 (5.75-89.75) | 192(48.5- 470.75) |
| Politician | 1IQR | 13 | 4 (30.8) | 5 (38.5) | 4 (30.8) | 5,480 (0.5) | 2.0 (1.0-11.0) | 7.0 (1.0-21.0) |
|  | 2IQR | 13 | 7 (53.8) | 6 (46.2) | 0 | 17,874 (1.6) | 1.0 (1.0-2.0) | 6.0 (2.0-12.0) |
|  | 3IQR | 12 | 5 (41.7) | 6 (50.0) | 1 (8.3) | 33,512 (3.1) | 2.5 (1.0-4.75) | 15.5 (2.75-18.25) |
|  | 4IQR | 13 | 5 (38.5) | 8 (61.5) | 0 | 1,035,150 (94.8) | 13.0 (12.0-32.0) | 62.0 (45.0-120.0) |

^a^ Percentages may not sum to 100 due to rounding.

^b^ RT = retweets
